# Resilience and Vulnerability to Trauma: Early Life Interventions Modulate Aversive Memory Reconsolidation in the Dorsal Hippocampus

**DOI:** 10.3389/fnmol.2019.00134

**Published:** 2019-05-28

**Authors:** Natividade de Sá Couto-Pereira, Carine Lampert, Aline dos Santos Vieira, Camilla Lazzaretti, Grasielle Clotildes Kincheski, Pablo Javier Espejo, Victor Alejandro Molina, Jorge Alberto Quillfeldt, Carla Dalmaz

**Affiliations:** ^1^Programa de Pós-graduação em Ciências Biológicas: Bioquímica, Instituto de Ciências Básicas da Saúde (ICBS), Universidade Federal do Rio Grande do Sul (UFRGS), Porto Alegre, Brazil; ^2^Programa de Pós-graduação em Neurociências, Instituto de Ciências Básicas da Saúde (ICBS), Universidade Federal do Rio Grande do Sul (UFRGS), Porto Alegre, Brazil; ^3^Departamento de Bioquímica, Instituto de Ciências Básicas da Saúde (ICBS), Universidade Federal do Rio Grande do Sul (UFRGS), Porto Alegre, Brazil; ^4^Instituto de Farmacología Experimental de Córdoba, Universidad Nacional de Cordoba (UNC), Cordoba, Argentina; ^5^Departamento de Biofísica, Instituto de Biociências, Universidade Federal do Rio Grande do Sul (UFRGS), Porto Alegre, Brazil

**Keywords:** neonatal handling, maternal separation, fear memory, reconsolidation, dorsal hippocampus, basolateral amygdala

## Abstract

Early life experiences program lifelong responses to stress. In agreement, resilience and vulnerability to psychopathologies, such as posttraumatic stress disorder (PTSD), have been suggested to depend on the early background. New therapies have targeted memory reconsolidation as a strategy to modify the emotional valence of traumatic memories. Here, we used animal models to study the molecular mechanism through which early experiences may later affect aversive memory reconsolidation. Handling (H)—separation of pups from dams for 10 min—or maternal separation (MS) — 3-h separation—were performed from PDN1–10, using non-handled (NH) litters as controls. Adult males were trained in a contextual fear conditioning (CFC) task; 24 h later, a short reactivation session was conducted in the conditioned or in a novel context, followed by administration of midazolam 3 mg/kg i.p. (mdz), known to disturb reconsolidation, or vehicle; a test session was performed 24 h after. The immunocontent of relevant proteins was studied 15 and 60 min after memory reactivation in the dorsal hippocampus (dHc) and basolateral amygdala complex (BLA). Mdz-treated controls (NH) showed decreased freezing to the conditioned context, consistent with reconsolidation impairment, but H and MS were resistant to labilization. Additionally, MS males showed increased freezing to the novel context, suggesting fear generalization; H rats showed lower freezing than the other groups, in accordance with previous suggestions of reduced emotionality facing adversities. Increased levels of Zif268, GluN2B, β-actin and polyubiquitination found in the BLA of all groups suggest that memory reconsolidation was triggered. In the dHc, only NH showed increased Zif268 levels after memory retrieval; also, a delay in ERK1/2 activation was found in H and MS animals. We showed here that reconsolidation of a contextual fear memory is insensitive to interference by a GABAergic drug in adult male rats exposed to different neonatal experiences; surprisingly, we found no differences in the reconsolidation process in the BLA, but the dHc appears to suffer temporal desynchronization in the engagement of reconsolidation. Our results support a hippocampal-dependent mechanism for reconsolidation resistance in models of early experiences, which aligns with current hypotheses for the etiology of PTSD.

## Introduction

Early life experiences modify the development of neural circuits, impacting the individuals on cognitive and emotional levels (Bolton et al., 2017) and may program the development of vulnerability or resilience to psychopathologies later in life (Franklin et al., 2012; Singh-Taylor et al., 2015; Di Segni et al., 2018). In fact, childhood adversities appear to be at the origin of a significant portion of mental disorders (Kessler et al., 2010), with particular relevance to posttraumatic stress disorder (PTSD). Stressful early life experiences have been proposed to induce an emotional dysregulation scenario that renders individuals more susceptible to develop this disorder after exposure to a traumatic event (Lanius et al., 2010). PTSD is a complex disorder that has lately been regarded as a memory disease (Van Marle, 2015); recent therapeutical approaches to PTSD have targeted reconsolidation as a strategy to modify the emotional valence of traumatic memories (Hartley and Phelps, 2010; Schiller et al., 2010; Akirav and Maroun, 2013; Kindt and van Emmerik, 2016; Dunbar and Taylor, 2017; Elsey and Kindt, 2017), but much more research is necessary to resolve conflicting results. In light of this, resistance to reconsolidation, a phenomenon that can be observed under certain conditions (Tronson and Taylor, 2007), might present both a challenge to the reconsolidation-based therapies, as well as a hypothetical pathological mechanism contributing to PTSD, since patients constantly and involuntarily evoke the traumatic memory but for some reason are incapable of modifying its emotional valence.

While the effects of early interventions on aversive memory acquisition, recall and extinction have been well studied in rodents (Wilber et al., 2009; Kosten et al., 2012; Diehl et al., 2014), to our knowledge, only one study has evaluated the effects of early stress on aversive memory reconsolidation (Villain et al., 2018), but in that study gestational and prepubertal stress was applied. The rodent neonatal period corresponds to early childhood in humans (Eiland and Romeo, 2013), an important period of the development that has been viewed as extremely relevant for the development of vulnerability or resilience to psychopathologies (Singh-Taylor et al., 2015); external interventions in this period, such as handling (H) or maternal separation (MS) modify the dynamics of dam-pup interaction in rats (Couto-Pereira et al., 2016), resulting in useful models to study the effects of early experiences on traumatic memories (Diehl et al., 2012, 2014).

Memory reconsolidation seems to comprise two distinct but entangled processes. First, the reactivated memory is destabilized and the trace becomes again labile; this process appears to depend on protein degradation *via* the ubiquitin-proteasome system—UPS, at least in the basolateral amygdala complex—BLA (Artinian et al., 2008; Lee et al., 2008; Jarome et al., 2011, 2016; Sol Fustiñana et al., 2014). NMDA receptors (NMDARs) activity is required for memory destabilization in the BLA, as shown by the administration of selective antagonists (Ben-Mamou et al., 2006; Milton et al., 2008). Further studies have shown that GluN2B-containing NMDARs are specifically involved with protein degradation *via* the UPS through activation of the calcium–calmodulin dependent protein kinase II (CaMKII), which in turn, activates the UPS (Mao et al., 2008; Jarome et al., 2016). The reconsolidation theory postulates that memory destabilization is followed by a restabilization phase that has been repeatedly shown to depend on protein synthesis (Nader et al., 2000; Pedreira et al., 2002; Artinian et al., 2008; Akirav and Maroun, 2013). Hence, activity-inducible transcription factors, such as Zif268, appear to be necessary for memory reconsolidation (Bozon et al., 2003; Maddox et al., 2011; Besnard et al., 2013).

Retrieval-induced labilization renders the memory susceptible to external or internal interferents, which may disrupt or update the original memory. Benzodiazepines (BZD), GABA_A_ receptor (GABA_A_R) positive allosteric modulators, have long been known for their amnestic properties (Malkani and Rosen, 2000), and their use as reconsolidation interferents has brought some interesting insights about the process (Makkar et al., 2010). In particular, midazolam (mdz), a rapid absorption BZD, has been applied in studies that focus on stress-modulatory effects on memory reconsolidation (Zhang and Cranney, 2008; Bustos et al., 2010; Ortiz et al., 2015; Espejo et al., 2016). These studies have shown that stress previous to training renders aversive memories resistant to reconsolidation (Bustos et al., 2010; Hoffman et al., 2015; Ortiz et al., 2015; Espejo et al., 2016), hypothetically by increasing memory strength, a feature that has been associated with decrease in NMDAR-mediated glutamatergic neurotransmission, particularly the GluN2B subunit (Wang et al., 2009), in the BLA (Ortiz et al., 2015; Espejo et al., 2016). These observations are in accordance with the essential role the amygdala plays in processing the emotional content of memories (LeDoux, 2003). In addition to the amygdala, the hippocampus, particularly its dorsal region—dorsal hippocampus (dHc), also has a relevant part in encoding and retrieving context-conditioned emotional memories (Phillips and LeDoux, 1992; Richter-Levin and Akirav, 2000). Both H and MS impact the development of the BLA and dHc, leading to morphological and functional changes in adulthood (Andersen and Teicher, 2004; Stevenson et al., 2009; Lajud et al., 2012; Diehl et al., 2014; Daskalakis et al., 2015; Koe et al., 2016).

Considering the long-term effects of neonatal interventions on emotionality and brain functioning, we hypothesized that H and MS adult rats could show changes in the reconsolidation of aversive memories, possibly resulting of alterations in signaling pathways, protein degradation and synaptic density dynamics associated with reconsolidation, in the BLA or dHc. Identifying mechanistic failures in the reconsolidation process may contribute to better understand the vulnerability to PTSD observed in individuals that suffered childhood adversities, as well as help improve reconsolidation-based therapies for the treatment of PTSD.

## Materials and Methods

### Subjects

All procedures were approved by the institutional Research Ethics Committee (CEUA-UFRGS #23844) and followed the Brazilian Law regarding the use of animals (Federal Law 11.794/2008) and the Guidelines for the Care and Use of Mammals in Neuroscience and Behavioral Research (National Research Council 2003). Care was taken to minimize animal suffering during the experiments.

Primiparous pregnant Wistar rats bred at our animal facility were used. At gestational day 17–18, they were single-housed in home cages made of Plexiglas (38 × 32 × 17 cm) with sawdust-covered floors and kept in a controlled environment (lights on from 7:00 to 19:00, temperature at 22 ± 2°C, standard rat chow and water provided *ad libitum*). The day of birth was considered postnatal day 0 (PND 0). All litters were randomly culled to 6–8 pups within 24 h after birth and were randomly assigned to one of the neonatal interventions described below. Weaning was performed on PND 21: males were randomly housed 3–4 per cage and remained under standard animal facility conditions until the beginning of the experiments.

To minimize the influence of each litter genetic load on our results, as adults, siblings were distributed among the various treatments: a maximum of two males from the same litter were assigned to the same drug or reactivation protocol for behavioral experiments and for biochemical experiments, only one male/litter was used in the same reactivation protocol. Females were assigned to other projects.

### Neonatal Intervention Models

Non-handled group (NH): pups and dams were left undisturbed until weaning, except for cage cleaning.

Neonatal Handling group (H): from PND 1–10, once a day, pups were gently placed together in a clean box lined with a paper towel, in a warm bath set to 32°C, where they remained for 10 min. After this period, pups were returned to their respective cages. This procedure was performed during the lights-on cycle, between 12:00 and 13:00.

Maternal separation group (MS): same protocol as the H group, except pups remained in the warm bath for 3 h and this intervention was conducted between 14:15 and 17:30.

Each litter had its own glove to be manipulated with, to avoid the spread of odors between nests. During the interventions, dams remained in the homecage, inside the room, so they could hear the pups’ vocalizations. From birth to weaning, cage cleaning was performed only when necessary, similarly for all groups: dirty sawdust was carefully removed from the cage, avoiding the nest area, and replaced with clean sawdust. Neither the dam nor the pups were disturbed during this process.

### Contextual Fear Conditioning

The contextual fear conditioning (CFC) task was performed on male rats, aged PND 90–100, that were subjected to the neonatal interventions described above. Experiments took place from 9:00 to 13:00. Two days before the beginning of the task, animals were taken to the room where they remained for 2 h, for acclimation; at the end of this period, they were gently handled and weighted: NH—397.3 ± 30.9; H—401.0 ± 36.7; MS—412.2 ± 38.2 [mean ± standard deviation; *F*_(2,99)_ = 1.302, *p* = 0.277, 1w-analysis of variance (ANOVA)].

CFC was performed in a wooden lidded apparatus (220 × 280 × 260 mm), with a transparent plastic wall, and a grid floor of parallel stainless steel bars 1.0 cm spaced (context A). In the training session, rats were allowed 3 min free exploration, to ensure they would form and consolidate a coherent representation of the context (Fanselow and Dong, 2010); animals then received three 1 s-duration footshocks of 0.8 mA, 30 s-interval, and 1 min later they were placed in clean homecages, to avoid contact with animals waiting to be trained. The electric current intensity was chosen based on a previous flinch-jump test (Couto-Pereira et al., 2016) and corresponds to an intensity to which NH, H and MS males all exhibited a jump response.

Two different Reactivation (React) sessions were conducted, 24 h after training. In the React A session, 55 animals (NH—20; H—17; MS—18) were re-exposed to context A, for 5 min. In the *pseudo* React B, 47 animals (NH—15; H—16; MS—16) were exposed to an unfamiliar context (B) for 5 min, which consisted of a plastic transparent box (400 × 220 × 260 mm) with smooth floor and walls, placed in a different room. Immediately after the end of both React sessions, animals received an intraperitoneal injection (i.p.) of either sterile saline solution—sal 1 mL/kg or mdz (“Dormonid,” Produtos Roche Químicos e Farmacêuticos, Brazil) diluted in sterile saline solution to a concentration of 3 mg/mL and administered at a dosage of 3 mg/kg. Twenty-four hours later, a Test session was conducted with all animals, in context A, for 5 min. All React and Test sessions were recorded; the training sessions of 39 animals (NH—13; H—13; MS—13) from the 15 min cyt experiment (described below) were recorded for baseline freezing assessment. Freezing duration, defined as the total absence of body and head movement except for that associated with breathing (Blanchard and Blanchard, 1969), was later scored by a single experimenter, whose analysis was compared with another experimenter for inter-reliability (Intraclass correlation coefficient = 0.988). Freezing is expressed in percentage of total session time.

### Biochemical Analyses

To evaluate the reconsolidation process at a molecular level, animals were euthanized at two different time points: 15 and 60 min after the end of a 5-min Reactivation session in the context A (React), with non re-exposed animals as controls (No React). No drugs were administered in this experiment. The 60 min post React experiment was divided into two experiments to obtain the cytosolic fraction (cyt) and, in a different subset of animals, a synaptosomal membrane-enriched fraction (synapt). A different subset of animals was euthanized at PND 90, without undergoing any experimental procedure except for the neonatal interventions (naïve). Adult male animals, weighing 381 ± 35 g (mean ± standard deviation), which were subjected to the neonatal interventions described above, were divided as follows: 15 min cyt—NH: 13 (React—6; No React—7), H: 13 (React—8; No React—5), MS: 14 (React—7; No React—7); 60 min cyt—NH: 16 (React—8; No React—8), H: 12 (React—6; No React—6), MS: 14 (React—6; No React—6); 60 min synapt—NH: 13 (React—6; No React—7), H: 12 (React—6; No React—6), MS: 11 (React—6; No React—5); naïve cyt—NH: 7; H: 8; MS: 6. Experiments were performed from 9:00 to 13:00.

#### Brain Dissection

Fresh brain tissue was dissected on ice. To dissect the dHc and BLA, coronal brain slices of 2 mm were cut using an acrylic brain matrix (#AL-1160, Alto). Structure boundaries were identified using a rat brain atlas (Paxinos and Watson, 1998): the dorsal portion of the hippocampus (dHc) was dissected from slices from bregma −2 mm to approximately bregma −5.52 mm and slices between approximately bregma −2 mm and −4 mm were used for the BLA, which was dissected using a 2 mm-diameter punch. Once dissected, samples were immediately frozen in liquid nitrogen and later stored at −80°C until further analysis.

#### Tissue Fractionation and Protein Extraction

##### Cyt Fraction

Samples were homogenized in 1:10 (w:v) hypotonic 10 mM Hepes buffer, containing 1.5 mM MgCl_2_, 10 mM KCl, 1 mM EDTA, 1 mM DTT, protease inhibitor (#11697498001, Roche, Germany) and phosphatase inhibitor (#88667, Pierce, ThermoFisher Scientific, Waltham, MA, USA) cocktails, pH = 7.9, 4°C. Samples were incubated on ice for 15 min to allow cell swelling; Nonidet P-40 0.6% (#E109, Amresco, Cleveland, OH, USA) was added and samples were placed on ice for 5 min more, with agitation every 15 s. Homogenates were centrifuged at 10,000 *g*, for 10 min, at 4°C, and the supernatant containing the cytosolic proteins was collected. Total protein content was determined using the PierceTM BCA Protein Assay kit (#23227, ThermoFisher Scientific, Waltham, MA, USA).

##### Synapt Fraction

Crude synaptosomal fractions (Synapt) of BLA and dHc were obtained based on previous studies (Dunah and Standaert, 2001; Jarome et al., 2011). This extraction method yields a fraction that is rich in synaptic membrane and synapse-associated proteins (Dunah and Standaert, 2001). Briefly, samples were thawed on ice and then homogeneized in 1:10 (m/v) TEVP+sucrose buffer [10 mM Tris-HCl, 5 mM NaF, 1 mM EDTA, 1 mM EGTA, phosphatase inhibitor cocktail (#88667, Pierce, ThermoFisher Scientific, Waltham, MA, USA), 320 mM sucrose, pH 7.4], using a pestle and a glass tissue grinder. Homogenates were centrifuged at 1,000 *g* for 10 min, at 4°C. The supernatant was collected and centrifuged at 10,000 *g* for 10 min, at 4°C. The resulting pellet was ressuspended in 1/5 detergent containing Lysis buffer [in 10 ml ultra-pure H_2_O: 0.0605 g Tris-HCl, 0.025 g sodium deoxycholate, 0.0876 g NaCl, 1 ml 10% SDS solution, protease inhibitor (#11697498001, Roche, Germany) and phosphatase inhibitor (#88667, Pierce, ThermoFisher Scientific, Waltham, MA, USA) cocktails] and then centrifuged at 15,000 *g* for 5 min, 4°C. The supernatant containing synaptosomal membrane proteins was collected and total protein content was determinedas above.

#### Western Blot

Denatured, reduced samples were loaded (40 μg protein/lane) on NuPAGE^®^ precast 4–12% gradient polyacrylamide gels (#NP0323BOX, ThermoFisher Scientific, Waltham, MA, USA), together with a 12–225 kDa molecular weight marker (#RPN800E, Amersham, GE Healthcare, UK). Electrophoresis and electrotransfer were performed on a XCell SureLock^®^ Mini-Cell and XCell II^TM^ Blot Module, respectively (#EI0002, Invitrogen, ThermoFisher Scientific, Waltham, MA, USA). Proteins were transferred to nitrocellulose membranes [1 h 50 at 50 V in transfer buffer (48 mM Trizma, 39 mM glycine, 20% methanol, 0.25% SDS)] and blots were then blocked for 2 h in Tris-buffered saline containing tween and 5% (m/v) non-fat dry milk or 5% (m/v) bovine serum albumin for phosphorylated proteins detection. Blots were incubated overnight, at 4°C, with one of the following primary antibodies: anti-Zif268 (1:1,000, #4154, Cell Signaling Technology, Danvers, MA, USA), anti-ERK 1/2 (1:4,000, #ABS44, Millipore, Germany), anti-pERK 1/2 (1:2,000, #9101, Cell Signaling Technology, Danvers, MA, USA), anti-GluN2A (1:1,000, #M264, Sigma-Aldrich, St. Louis, MO, USA), anti-GluN2B (1:2,000, #06600, Millipore, Germany), anti-pGluN2B (1:1,000, #M2442, Sigma-Aldrich, USA), anti-GABA_A_R α1–6 (1:500, #sc-376282, Santa Cruz Biotechnology, Santa Cruz, CA, USA), anti-synaptophysin (1:2,000, #AB9272, Millipore, Germany), anti-ubiquitin k48-specific (1:500, #05–1307, Merck-Millipore, Germany), anti-α-tubulin (1:4,000, #T6074, Sigma-Aldrich, St. Louis, MO, USA) or anti-β-actin (1:3,500, #8457, Cell Signaling Technology, Danvers, MA, USA). Secondary peroxidase-conjugated anti-rabbit antibody (1:1,000, #AP132P, Merck-Millipore, Germany, or 1:2,500, Jackson ImmunoResearch, West Grove, PA, USA) or anti-mouse antibody (1:1,000, #402335, Calbiochem, Merck-Millipore, Germany) was incubated for 2 h at room temperature. Blots were developed using a chemiluminescence ECL kit (#RPN2209, Amersham, GE Healthcare, UK) and images were digitally acquired using ImageQuant LAS 4,000 (GE Healthcare Bio-Sciences AB, Umeå, Sweden); in the k48-linked polyubiquitination experiment, chemiluminescence was acquired on x-ray films. Antibody stripping was performed using 1 M sodium hydroxide and stripping efficiency was confirmed by incubating blots with the respective secondary antibody, followed by chemiluminescence detection.

Optical density (OD) was determined using the software ImageJ (National Institutes of Health, Bethesda, MD, USA). Results were quantified as the ratio of the protein of interest OD to that of the loading control. β-actin was used as loading control for all western blot experiments except in BLA synaptosomes blots, in which α-tubulin was used, as explained in the Results section. Loading control absolute OD was tested in all experiments for differences between groups and was only accepted if no significant interaction or main effects were found (*p* > 0.05, 2w-ANOVA, data not shown). All results are expressed in percentage of control (NH No Reactivation group).

### Statistical Analysis

Data were analyzed using the software SPSS version 16.0. Levene’s test of equality of error variances was used to test the homogeneity of group variances. Two-way analysis of variance (2w-ANOVA), with neonatal intervention and drug as factors, or one-way ANOVA (1w-ANOVA), with neonatal intervention as factors were performed for behavioral results. 2w-ANOVA with neonatal intervention and reactivation as factors was used for all biochemical results, except for the analysis of naïve animals, in which a 1w-ANOVA was used. Tukey *post hoc* test was performed to compare groups, when appropriate. Data are expressed as mean ± standard error of the mean (SEM). Statistical significance was set at *p* < 0.05. Data was excluded from behavioral experiments only if freezing was more than 2 standard deviations from the group mean. A total of four animals was excluded using this criteria: 2 NH, 1 H and 1 MS.

## Results

### Mdz Disrupts Memory Reconsolidation in NH but Not in H and MS Adult Male Rats

**Figure 1:**
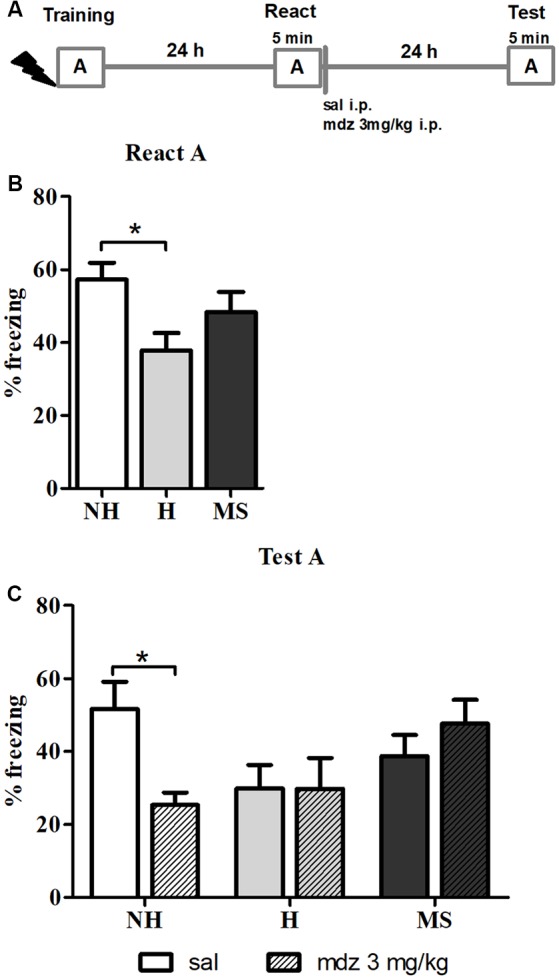
Effect of midazolam (mdz) injection after memory reactivation by re-exposure to context A, in adult male rats that were non-handled (NH) or subjected to handling (H) or maternal separation (MS) in the neonatal period. Only NH animals were sensitive to the disrupting effect of mdz on reconsolidation. **(A)** Schematic diagram of the experimental design. **(B)** Freezing in context A, in the Reactivation (React) session, *n* = 17–20/group. **(C)** Freezing in context A, in the Test session, of animals that received either sal or mdz 3 mg/kg i.p. after the React session, *n* = 8–10/group. Data are expressed as mean ± standard error of the mean (SEM), as percentage of total session duration. Two-way analysis ofvariance (2w-ANOVA) was used for statistical analyses; *p < 0.05. Statistics results are presented in detail in subsections “Mdz Disrupts Memory Reconsolidation in NH but Not in H and MS Adult Male Rats” and “H Animals Exhibit Less Freezing When Re-exposed to the Conditioned Context.”

To study the effects of different neonatal interventions on aversive memory reconsolidation, we first established a protocol that triggered contextual fear memory reconsolidation in male adult rats. We used mdz, a GABAergic drug known to interfere with memory reconsolidation (Bustos et al., 2006), to provide behavioral evidence that the protocol used was inducing reconsolidation, by testing animals 24 h later in context A, for 5 min (Figure 1A). A significant interaction between neonatal intervention and drug was found for freezing, in the Test session (*F*_(2,48)_ = 4.108, *p* = 0.023, 2w-ANOVA); no significant main effects were found (neonatal intervention: *F*_(2,48)_ = 2.012, *p* = 0.145; drug: *F*_(2,48)_ = 1.143, *p* = 0.290; 2w-ANOVA). *Post hoc* analysis revealed that NH animals that were administered mdz 3 mg/kg after React had freezing levels significantly lower than NH rats that received sal (*p* = 0.040, Tukey *post hoc* test), showing that mdz administered during the reconsolidation window successfully disrupted memory in NH rats and providing evidence that the experimental conditions employed here successfully induced memory reconsolidation in control animals (Figure 1C).

Furthermore, neonatal interventions appeared to affect this process; there were no differences in freezing between sal or mdz-treated H and MS rats (*p* = 1.000 and *p* = 0.937, respectively; Tukey *post hoc* test), suggesting that fear memory in these animals was resistant to interference by a GABA_A_R positive allosteric modulator.

### H Animals Exhibit Less Freezing When Re-exposed to the Conditioned Context

In the React A session (Figure 1B), a significant effect of neonatal intervention was found (*F*_(2,52)_ = 3.448, *p* = 0.039, 1w-ANOVA); Tukey *post hoc* revealed that H animals exhibited significantly less freezing than NH (*p* = 0.030), but no differences were detected between MS and NH rats (*p* = 0.516) or MS and H rats (*p* = 0.325), which is in accordance with previous studies on aversive memory consolidation in H and MS rats (Meerlo et al., 1999; Ladd et al., 2004; Kosten et al., 2006; Diehl et al., 2007; Arnett et al., 2015).

Context-induced freezing may be influenced by the emotional valence of the aversive experience or by pain sensitivity. Regarding training strength, we have previously reported that 0.8 mA is an electric current intensity that generates similar behavioral responses and corticosterone secretion levels after conditioned context exposure in NH, H and MS male rats; also, no differences on footshock sensitivity were observed in these groups in the flinch-jump test (Couto-Pereira et al., 2016).

### MS Rats Generalize the Fear Response to Novel Environments

**Figure 2:**
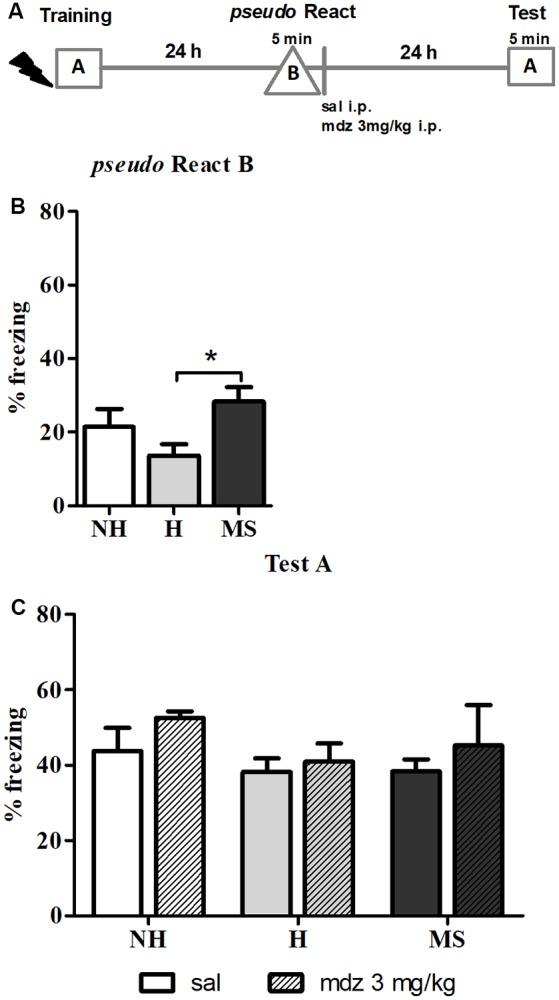
The effect of midazolam (mdz) injection after a *pseudo-*reactivation in context B was tested by re-exposure to context A, in adult male rats that were non-handled (NH) or subjected to handling (H) or maternal separation (MS) in the neonatal period. Mdz effect on reconsolidation is specific to reactivated memories. **(A)** Schematic diagram of the experimental design. **(B)** Freezing in context B, in the *pseudo* Reactivation (React) session, *n* = 15–17/group. **(C)** Freezing in context A, in the Test session, of animals that received either sal or mdz 3 mg/kg after the *pseudo* React B session, *n* = 7–9/group. Data are expressed as mean ± SEM, in percentage of total session duration. 2w-ANOVA was used for statistical analyses; *p < 0.05. Statistics results are presented in detail in subsections “MS Rats Generalize the Fear Response to Novel Environments” and “Mdz Disrupting Effect on Reconsolidation Requires Properly Reactivated Memories.”

Memory precision was tested by exposing the animals to an unfamiliar context 24 h after training –*pseudo* React in context B (Figure 2B). A significant effect of neonatal intervention was detected (*F*_(2,42)_ = 4.102, *p* = 0.027, 1w-ANOVA); Tukey *post hoc* analysis showed that MS rats freezing was significantly increased in the new context compared to H animals (*p* = 0.020), suggesting that MS induces generalization of fear memory, at least in comparison to H; no differences were found between H and MS rats compared to controls (*p* = 0.339 and *p* = 0.448, respectively).

Regarding this result, anxiety could be a confounding factor, since several studies reported that MS animals show increased anxiety-like behaviors in unfamiliar environments (Romeo et al., 2003; de Kloet et al., 2005; Makena et al., 2012); hence, freezing in context B could result of novelty-induced anxiety. To test this hypothesis, we examined freezing in the training session, during the 3 min pre-shock, when animals were exploring a context that was new to them at that moment. Consistent with our previous report (Diehl et al., 2014), before conditioning, neonatal interventions did not change freezing in response to a new environment: NH—1.3% ± 0.5, H—0.9% ± 0.5, MS—1.0% ± 0.5 (*n* = 13/group). Since no differences were found at this point (*F*_(2,36)_ = 0.224, *p* = 0.801, 1w-ANOVA), it is valid to assume that the aversive experience was necessary to induce the generalization of fear to unconditioned environments observed in MS rats.

### Mdz Disrupting Effect on Reconsolidation Requires Properly Reactivated Memories

The *pseudo* React B session was also performed to evaluate the specificity of the effect of mdz on memory reconsolidation. After exposure to context B, sal or mdz 3 mg/kg i.p. was injected and 24 h later, animals were re-exposed to the conditioned context (A) for 5 min (Figures 2A,C). No significant interaction or main effects were detected (interaction: *F*_(2,39)_ = 0.143, *p* = 0.867; neonatal intervention: *F*_(2,39)_ = 1.155, *p* = 0.326; drug: *F*_(1,39)_ = 1.474, *p* = 0.232; 2w-ANOVA). Consistently with previous reports (Bustos et al., 2006, 2009), mdz only impaired memory when it was properly reactivated in the conditioned context (A), providing further evidence of the specific effect of the drug in disrupting memory reconsolidation after retrieval.

### ERK 1/2 Activity and Zif268 Levels Were Not Changed in the BLA 15 min After Aversive Memory Reactivation

**Figure 3:**
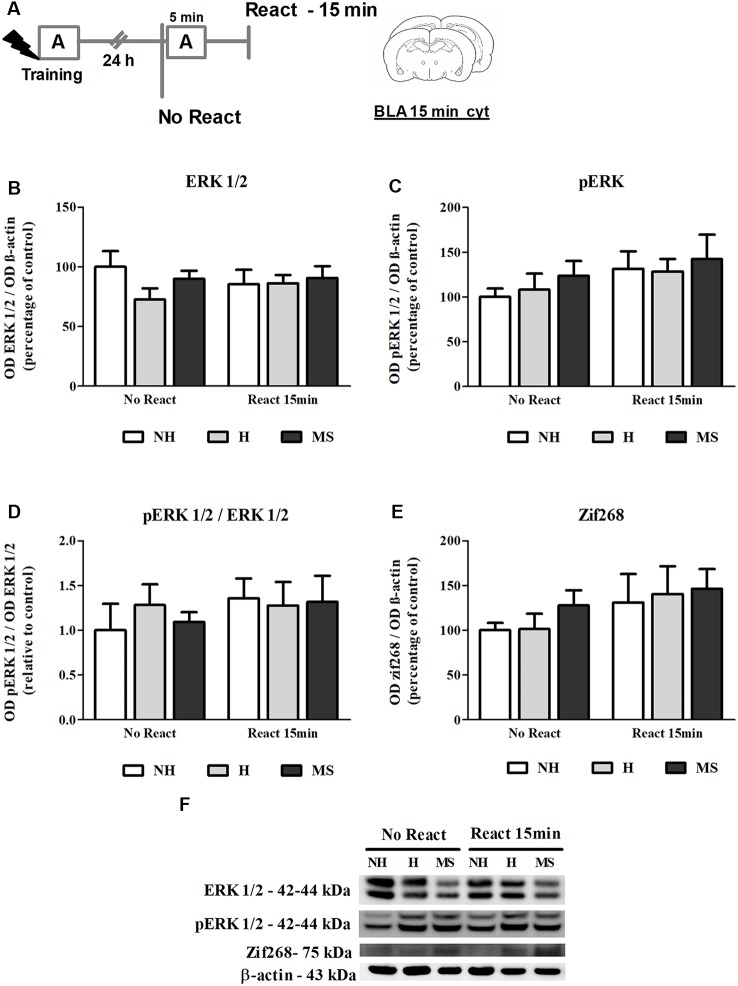
ERK 1/2, pERK 1/2 and Zif268 immunocontent in the basolateral amygdala complex (BLA) cytosolic fraction (cyt) of adult male rats that were non-handled (NH) or subjected to handling (H) or maternal separation (MS) in the neonatal period, 15 min after Reactivation (React) compared to trained animals that were not re-exposed to the training context (No React). No significant differences in ERK 1/2 activation or Zif268 expression were found in the BLA at this time point. **(A)** Schematic diagram of the experimental design; **(B)** ERK 1/2; **(C)** pERK 1/2; **(D)** calculated ratio of pERK 1/2 per ERK 1/2 immunocontent; **(E)** Zif268; **(F)** representative Western blot bands. Data are expressed as mean ± SEM. *n* = 5–7/group. 2w-ANOVA was used for statistical analyses. Statistics results are presented in detail in subsection “ERK 1/2 Activity and Zif268 Levels Were not Changed in the BLA 15 min After Aversive Memory Reactivation.”

To better understand the resistance to reconsolidation interference in H and MS rats, a different subset of animals was trained and re-exposed to the conditioned apparatus A (React) and 24 h later dHc and BLA were collected 15 or 60 min after the end of the session; since the goal of this set of experiments was to evaluate biochemical changes during reconsolidation induced by reactivation, animals that were trained but not re-exposed to the context (No React) were used as controls (Figure 3A).

ERK 1/2 levels, as well as its active form phosphorylated at residues T202/Y204, were evaluated by Western blot, 15 min after the end of the Reactivation session, in the BLA of NH, H and MS adult males (Figures 3B–D,F). No significant interaction or main effects were found for ERK 1/2 or its phosphorylated form (ERK 1/2: interaction—*F*_(2,33)_ = 0.929, *p* = 0.405; neonatal intervention—*F*_(2,33)_ = 0.946, *p* = 0.399; reactivation—*F*_(1,33)_ < 0.001, *p* = 0.991; pERK 1/2: interaction: *F*_(2,33)_ = 0.074, *p* = 0.929; neonatal intervention—*F*_(2,33)_ = 0.576, *p* = 0.568; reactivation—*F*_(1,33)_ = 2.465, *p* = 0.127; 2w-ANOVA). BLA ERK 1/2 activation happens later than in the dHc, reaching a peak at 30 min post-reactivation (Besnard et al., 2014). Similarly, we did not find any significant differences in Zif268 levels at this time point (Figures 3E,F; interaction: *F*_(2,30)_ = 0.107, *p* = 0.899; neonatal intervention: *F*_(2,30)_ = 0.516, *p* = 0.602; reactivation: *F*_(1,30)_ = 2.516, *p* = 0.123; 2w-ANOVA).

### Zif268 Levels Increase in the BLA, 60 min After Aversive Memory Reactivation

**Figure 4:**
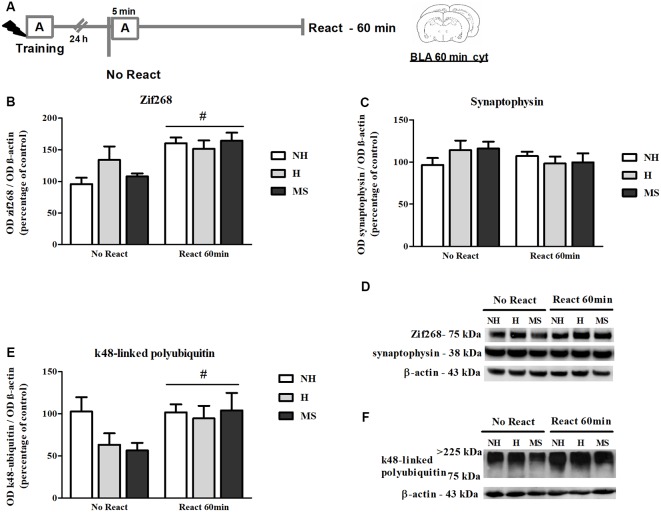
Zif268, synaptophysin and k48-linked polyubiquitinated proteins immunocontent in the basolateral amygdala complex (BLA) cytosolic fraction (cyt) of adult male rats that were non-handled (NH) or subjected to handling (H) or maternal separation (MS) in the neonatal period, 60 min after Reactivation (React) compared to trained animals that were not re-exposed to the training context (No React). Memory reactivation induced a significant increase in Zif268 and polyubiquitinated proteins in the BLA cyt of all groups. **(A)** Schematic diagram of the experimental design; **(B)** Zif268; **(C)** synaptophysin; **(D)** representative Western blot bands; **(E)** k48-linked polyubiquitin proteins; **(F)** representative Western blot bands. Data are expressed as mean ± SEM. *n* = 5–8/group. 2w-ANOVA was used for statistical analyses; ^#^*p* < 0.05 (main effect of reactivation). Statistics results are presented in detail in subsections “Zif268 Levels Increase in the BLA, 60 min After Aversive Memory Reactivation,” “Memory Reactivation Induces Changes in Receptor Composition at the BLA Synapses” and “Synaptic NMDA and GABA_A_R Subunits Were not Changed by Memory Reactivation in the dHc.”

The immunocontent of Zif268 was also analyzed 60 min post-reactivation (Figures 4A,B,D), a time point at which higher increases in the BLA have been reported (Besnard et al., 2014). In fact, we found significantly increased levels of Zif268 in NH, H and MS rats that were exposed to context A, compared to No React controls (*F*_(1,35)_ = 19.965, *p* < 0.01, 2w-ANOVA, main effect of Reactivation). Zif268 induction has been implicated in the memory reconsolidation process in several studies (Hall et al., 2001; Bozon et al., 2003; Lee et al., 2004; Maddox et al., 2011; Besnard et al., 2013; Espejo et al., 2016). No interaction or main effect of neonatal intervention were found (interaction: *F*_(2,35)_ = 1.995, *p* = 0.151; neonatal intervention: *F*_(2,35)_ = 0.761, *p* = 0.475; 2w-ANOVA).

### Memory Reactivation Induces Changes in Receptor Composition at the BLA Synapses

**Figure 5:**
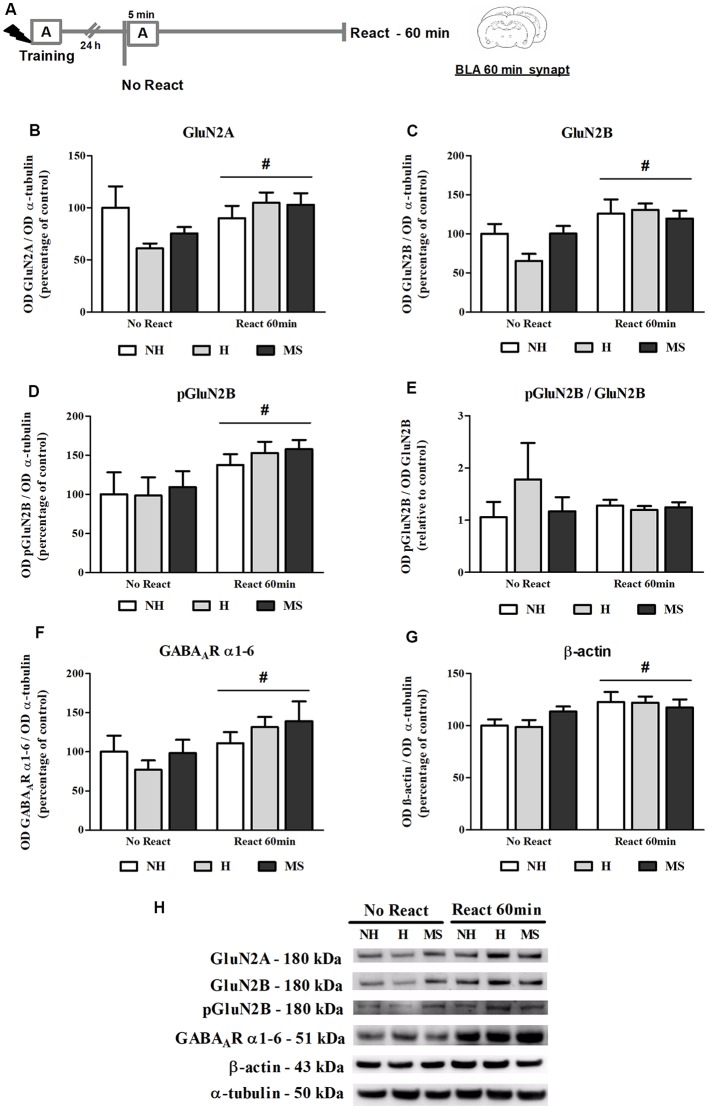
NMDA receptor (NMDAR) subunits GluN2A and GluN2B (total and phosphorylated), GABA_A_R α1–6 subunits and β-actin immunocontent in the basolateral amygdala complex (BLA) synaptosome membrane fraction (synapt) of adult male rats that were non-handled (NH) or subjected to handling (H) or maternal separation (MS) in the neonatal period, 60 min after Reactivation (React) compared to trained animals that were not re-exposed to the training context (No React). Memory reactivation induced a significant increase in all receptor subunits and also β-actin in the BLA synapt of all groups. **(A)** Schematic diagram of the experimental design; **(B)** GluN2A; **(C)** GluN2B; **(D)** pGluN2B; **(E)** calculated ratio of pGluN2B per GluN2B immunocontent; **(F)** GABA_A_R α1–6 subunits; **(G)** β-actin; **(H)** representative Western blot bands. Data are expressed as mean ± SEM. *n* = 5–7/group. 2w-ANOVA was used for statistical analyses. ^#^*p* < 0.05 (main effect of reactivation). Statistics results are presented in detail in subsection “Memory Reactivation Induces Changes in Receptor Composition at the BLA Synapses.”

NMDAR subunits GluN2A and GluN2B (total and phosphorylated form), GABA_A_R subunits α1–6 and β-actin were analyzed in the BLA synapt fraction, 60 min post-reactivation (Figure 5A).

The immunocontent of GluN2B, pGluN2B, GluN2A and GABA_A_R α1–6 subunits was significantly increased in the synapt fraction by memory reactivation (GluN2B: *F*_(1,25)_ = 12.861, *p* = 0.001; pGluN2B: *F*_(1,25)_ = 8.311, *p* = 0.009; GluN2A: *F*_(1,25)_ = 4.225, *p* = 0.050; 2w-ANOVA, main effect of reactivation). For GluN2A, a trend towards an interaction was also found (*F*_(2,25)_ = 2.575, *p* = 0.098, 2w-ANOVA). No significant interactions or main effects of neonatal intervention were found for the other receptor subunits analyzed here (GluN2B: interaction—*F*_(2,25)_ = 1.909, *p* = 0.169; neonatal intervention—*F*_(2,25)_ = 0.764; *p* = 0.476; pGluN2B: interaction—*F*_(2,25)_ = 0.095, *p* = 0.910; neonatal intervention—*F*_(2,25)_ = 0.287; *p* = 0.753; GluN2A: neonatal intervention—*F*_(2,25)_ = 0.505; *p* = 0.610; 2w-ANOVA). Phosphorylation of GluN2B subunit was assessed at its major phosphorylation site, Y1472 (Chen and Roche, 2007). While both total and phosphorylated forms of GluN2B were significantly increased by reactivation, as described above, no interaction or main effects were found for the ratio pGluN2B/GluN2B (interaction: *F*_(2,25)_ = 0.978, *p* = 0.392; neonatal intervention: *F*_(2,25)_ = 0.643, *p* = 0.536; reactivation: *F*_(1,25)_ = 0.146, *p* = 0.706; 2w-ANOVA), which means that the increase in GluN2B at synapses was accompanied by its phosphorylation (Figures 5B–E,H).

A ratio of the synapt immunocontent of GluN2A/GluN2B was also calculated since it has been shown that increased GluN2A/GluN2B synaptic ratio in the BLA inhibits retrieval-dependent memory destabilization (Holehonnur et al., 2016). No significant interaction or main effects were found for this ratio (interaction: *F*_(2,25)_ = 0.965, *p* = 0.395; neonatal intervention: *F*_(2,25)_ = 1.978, *p* = 0.159; reactivation: *F*_(1,25)_ = 0.447, *p* = 0.510; 2w-ANOVA, data not shown).

Since a GABA_A_R positive allosteric modulator was used as a memory interferent in this work, it was important to assess this receptor concentration at synapses. To do so, we used an antibody that recognizes all 1–6 α-type subunits of the GABA_A_R. Since all GABA_A_R possess two α subunits in their composition (Olsen and Sieghart, 2009), measurement of total levels of this subunit should provide an approximate determination of total receptor content. While a reactivation effect was observed (*F*_(1,30)_ = 5.815, *p* = 0.022; 2w-ANOVA), similarly to NMDAR subunits, no interaction or neonatal intervention effect was observed for this measure (interaction: *F*_(2,30)_ = 0.818, *p* = 0.451; neonatal intervention: *F*_(2,30)_ = 0.382, *p* = 0.686; 2w-ANOVA), suggesting that GABA_A_R levels were not altered in the BLA synapses of H, NH and MS rats before exposure to context (Figures 5F,H). This does not necessarily represent basal levels since No Reactivation rats had been trained in an aversive task 24 h earlier.

A significant reactivation-induced increase in β-actin was also found for all neonatal treatments (*F*_(1,30)_ = 8.354, *p* = 0.007, 2w-ANOVA, main effect of Reactivation; Figures 5G–H); this effect is in accordance with previous reports that show that actin filaments proliferation is necessary for reconsolidation (Rehberg et al., 2010; Lamprecht, 2016; Lunardi et al., 2018; Popik et al., 2018). No significant interaction (*F*_(2,30)_ = 1.178, *p* = 0.322, 2w-ANOVA) or main effect of neonatal intervention (*F*_(2,30)_ = 0.274, *p* = 0.762, 2w-ANOVA) were found. To detect a possible confounding effect on our BLA synapt results that could arise from a variation in the number or activity of synapses in our neonatal intervention groups, we measured synaptophysin levels in the BLA cyt fraction, a protein that is commonly used as pre-synaptic terminal marker (Andersen and Teicher, 2004). No significant interaction or main effects were found concerning synaptophysin levels in the BLA of NH, H and MS adult rats (Figures 4C,D; interaction: *F*_(2,35)_ = 1.861, *p* = 0.171; neonatal intervention: *F*_(2,35)_ = 0.281, *p* = 0.757; reactivation: *F*_(1,35)_ = 1.047, *p* = 0.313; 2w-ANOVA).

**Figure 6:**
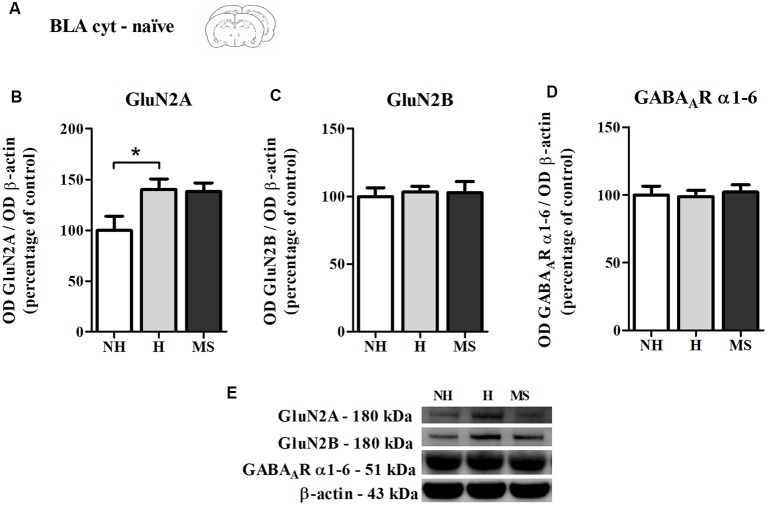
NMDAR subunits GluN2A and GluN2B and GABA_A_R α1–6 subunits immunocontent in the basolateral amygdala complex (BLA) cytosolic fraction (cyt) of adult male rats that were non-handled (NH) or subjected to handling (H) or maternal separation (MS) in the neonatal period. **(A)** Schematic diagram of the experimental design; **(B)** GluN2A; **(C)** GluN2B; **(D)** GABA_A_R α1–6 subunits; **(E)** representative Western blot bands. Data are expressed as mean ± SEM. *n* = 6–8/group. 1w-ANOVA was used for statistical analyses. *p < 0.05. Statistics results are presented in detail in subsection “Memory Reactivation Induces Changes in Receptor Composition at the BLA Synapses.”

Also as a control experiment, we analyzed the immunocontent of GluN2A, GluN2B and GABA_A_R subunits α1–6 in the BLA cyt fraction of naïve NH, H and MS rats, to check for differences which could influence our results (Figure 6A). NH animals showed a higher basal level of GluN2A compared to NH (*F*_(2,17)_ = 4.045, *p* = 0.037; 1w-ANOVA; Tukey *post hoc*: NH vs. H: *p* = 0.044, NH vs. MS: *p* = 0.08; Figures 6B,E). This difference disappeared after training in the CFC paradigm, as can be seen by the H No React result (Figure 5B), although no direct comparison was made between these data. No significant differences were detected for GluN2B (*F*_(2,17)_ = 0.084, *p* = 0.919; 1w-ANOVA; Figures 6C,E), GABA_A_R subunits α1–6 (*F*_(2,18)_ = 0.093, *p* = 0.912; 1w-ANOVA; Figures 6D,E) or the ratio GluN2A/GluN2B (*F*_(2,16)_ = 1.724, *p* = 0.210; 1w-ANOVA; data not shown).

### Neonatal Interventions Change ERK 1/2 Activation in the dHc, 15 min After Aversive Memory Reactivation

**Figure 7:**
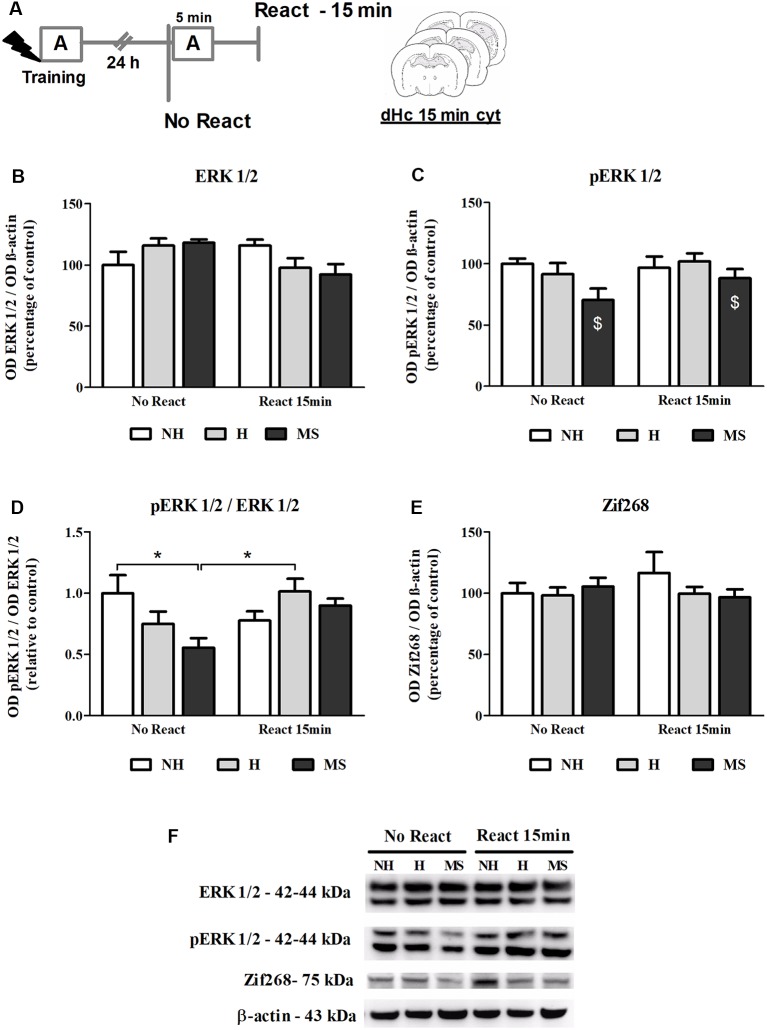
ERK 1/2, pERK 1/2 and Zif268 immunocontent in the dorsal hippocampus (dHc) cytosolic fraction (cyt) of adult male rats that were non-handled (NH) or subjected to handling (H) or maternal separation (MS) in the neonatal period, 15 min after Reactivation (React) compared to trained animals that were not re-exposed to the training context (No React). Activation of ERK 1/2 in the dHc appears to be delayed, particularly in MS rats. **(A)** Schematic diagram of the experimental design; **(B)** ERK 1/2; **(C)** pERK 1/2; **(D)** calculated ratio of pERK 1/2 per ERK 1/2 immunocontent; **(E)** Zif268; **(F)** representative Western blot bands. Data are expressed as mean ± SEM. *n* = 5–8/group. 2w-ANOVA was used for statistical analyses. *p < 0.05; ^$^*p* < 0.05 (main effect of neonatal intervention). Statistics results are presented in detail in subsection “Neonatal Interventions Change ERK 1/2 Activation in the dHc, 15 min After Aversive Memory Reactivation.”

ERK 1/2 levels, as well as its activation by phosphorylation, were also studied in the dHc, 15 min post-reactivation (Figure 7A).

A significant interaction was found for ERK 1/2 levels in this brain region (*F*_(2,31)_ = 3.905, *p* = 0.031, 2w-ANOVA; Figures 7B,F) but no significant main effects were found (neonatal intervention: *F*_(2,31)_ = 0.059, *p* = 0.942; reactivation: *F*_(2,31)_ = 2.142, *p* = 0.153; 2w-ANOVA). Tukey *post hoc* analysis revealed no significant differences between groups (*p* > 0.05). Still, the significant interaction between neonatal intervention and reactivation allows us to infer that early after memory reactivation, total ERK 1/2 appears to decrease in H and MS, but increase in NH animals. Phosphorylated ERK 1/2 (pERK 1/2) levels were lower in MS animals, independently of reactivation (*F*_(2,31)_ = 3.589, *p* = 0.040; 2w-ANOVA, main effect of neonatal intervention; Figures 7C,F); no interaction (*F*_(2,31)_ = 0.926, *p* = 0.407; 2w-ANOVA) or main effect of reactivation (*F*_(1,31)_ = 1.697, *p* = 0.202; 2w-ANOVA) were found. A ratio of pERK 1/2 per total ERK 1/2 levels was calculated to evaluate changes in the relative phosphorylation status of ERK 1/2. A significant interaction was found for this ratio (*F*_(2,31)_ = 4.590, *p* = 0.018, 2w-ANOVA; Figures 7D,F): Tukey *post hoc* test showed that cytosolic ERK 1/2 activation levels were lower in the dHc of MS No React rats, compared to NH No React (*p* = 0.049) and H React (*p* = 0.030); but MS React animals were not different from other React groups (vs. NH React—*p* = 0.977; vs. H React—*p* = 0.994), which reveals a great increase in ERK 1/2 phosphorylation at this time point, when NH animals already stabilized this process (NH No React vs. NH React—*p* = 0.630). No main effects of neonatal intervention (*F*_(2,31)_ = 1.596, *p* = 0.219; 2w-ANOVA) or reactivation (*F*_(1,31)_ = 2.426, *p* = 0.130; 2w-ANOVA) were found for this variable.

Increases in Zif268 immunocontent in hippocampal areas have been reported as early as 15 min post-reactivation (Besnard et al., 2014). Here, we did not find any significant differences in Zif268 levels at this time point (Figures 7E,F; interaction: *F*_(2,31)_ = 0.924, *p* = 0.407; neonatal intervention: *F*_(2,31)_ = 0.547, *p* = 0.584; reactivation: *F*_(1,31)_ = 0.154, *p* = 0.697; 2w-ANOVA). The lack of a significant effect may be attributed to the lower sensitivity of the Western blot technique compared to immunohistochemistry, which was used in the aforementioned study.

### Zif268 Levels Increase in the dHc of NH, but Not H or MS, 60 min After Aversive Memory Reactivation

**Figure 8:**
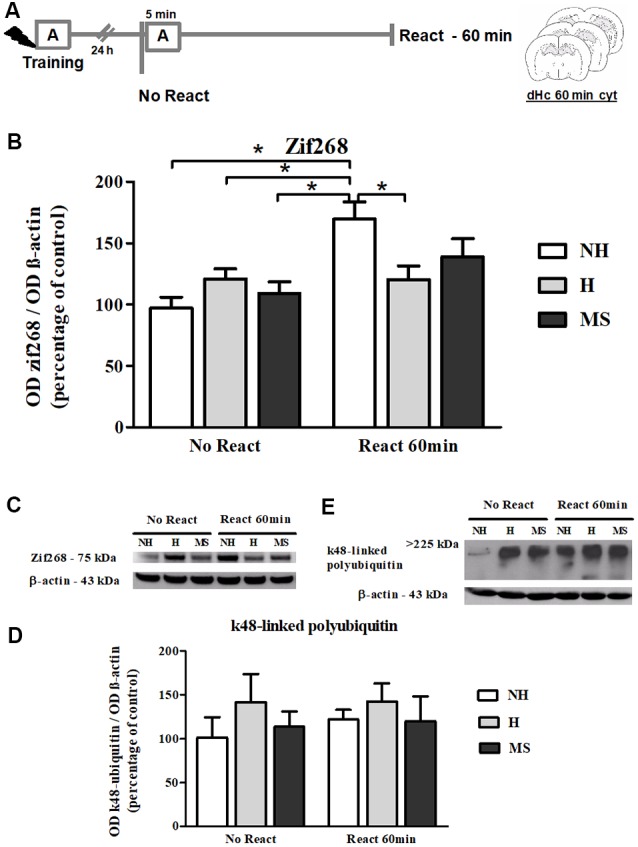
Zif268 and k48-linked polyubiquitinated proteins immunocontent in the dHc cytosolic fraction (cyt) of adult male rats that were non-handled (NH) or subjected to handling (H) or maternal separation (MS) in the neonatal period, 60 min after Reactivation (React) compared to trained animals that were not re-exposed to the training context (No React). Only NH rats exhibited the expected increase in Zif268 in the dHc induced by memory reactivation. **(A)** Schematic diagram of the experimental design; **(B)** Zif268; **(C)** representative Western blot bands; **(D)** k48-linked polyubiquitin proteins; **(E)** representative Western blot bands. Data are expressed as mean ± SEM. *n* = 6–8/group. 2w-ANOVA was used for statistical analyses; *p < 0.05. Statistics results are presented in detail in subsections “Zif268 Levels Increase in the dHc of NH, but Not H or MS, 60 min After Aversive Memory Reactivation” and “k48-Linked Polyubiquitin Levels Were Increased by Reactivation in the BLA, but Not in dHc.”

Zif268 levels steadily increase in dentate gyrus during the reconsolidation window (Besnard et al., 2014). Hence, we also evaluated Zif268 immunocontent at 60 min post-reactivation (Figures 8A–C). A significant interaction was found (*F*_(2,29)_ = 5.361, *p* = 0.010; 2w-ANOVA); Tukey *post hoc* showed that memory reactivation in NH induced a significant increase in the immunocontent of this transcription factor, compared to NH No React (*p* = 0.001), H No React (*p* = 0.042), H React (*p* = 0.005) and MS No React (*p* = 0.011). React H and MS were not different from their respective No React controls (H: *p* = 1.000; MS: *p* = 0.558). A significant effect of Reactivation was also encountered (*F*_(1,29)_ = 12.554, *p* = 0.001; 2w- ANOVA), but there was no significant effect of neonatal intervention on this variable (*F*_(2,29)_ = 0.690, *p* = 0.509; 2w- ANOVA).

### Synaptic NMDA and GABA_A_R Subunits Were Not Changed by Memory Reactivation in the dHc

**Figure 9:**
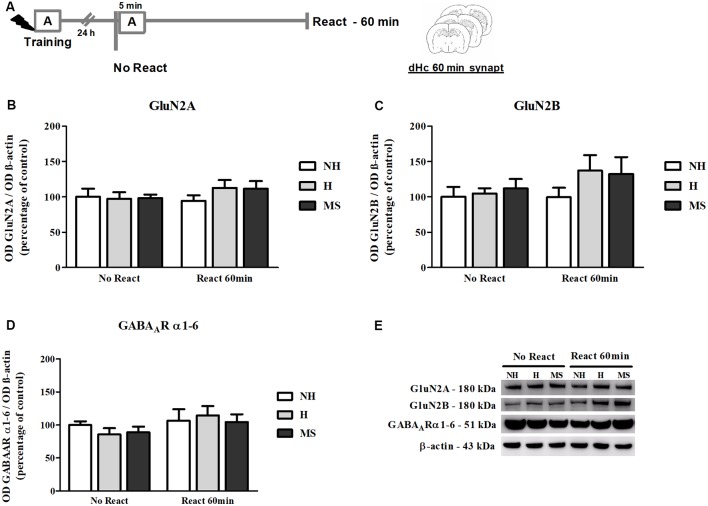
NMDAR subunits GluN2A and GluN2B and GABA_A_R α1–6 subunits immunocontent in the dHc synaptosome membrane fraction (synapt) of adult male rats that were non-handled (NH) or subjected to handling (H) or maternal separation (MS) in the neonatal period, 60 min after Reactivation (React) compared to trained animals that were not re-exposed to the training context (No React). No significant changes in N-Methyl-D-aspartate (NMDA) or GABA_A_R subunit composition were found in the dHc. **(A)** Schematic diagram of the experimental design; **(B)** GluN2A; **(C)** GluN2B; **(D)** GABA_A_R α1–6 subunits; **(E)** representative Western blot bands. Data are expressed as mean ± SEM. *n* = 5–7/group. 2w-ANOVA was used for statistical analyses. Statistics results are presented in detail in subsection “Synaptic NMDA and GABA_A_R Subunits Were not Changed by Memory Reactivation in the dHc.”

NMDAR subunits GluN2A and GluN2B and α1–6 subunits of the GABA_A_R were also measured in the dHc synapt fraction, 60 min post-reactivation (Figure 9A).

No significant changes were found in the immunocontent of subunits GluN2A (interaction: *F*_(2,30)_ = 0.679, *p* = 0.515; neonatal intervention: *F*_(2,30)_ = 0.395, *p* = 0.677; reactivation: *F*_(1,30)_ = 0.895, *p* = 0.352, 2w-ANOVA; Figures 9B,E) or GluN2B (interaction: *F*_(2,30)_ = 0.508, *p* = 0.607; neonatal intervention: *F*_(2,30)_ = 1.144, *p* = 0.332; reactivation: *F*_(1,30)_ = 1.604, *p* = 0.215, 2w-ANOVA; Figures 9C,E). For GABA_A_R α1–6 subunits, a trend towards a main effect of reactivation was found (*F*_(1,30)_ = 3.074, *p* = 0.090; 2w-ANOVA; Figures 9D,E), but no significant interaction (*F*_(2,30)_ = 0.148, *p* = 0.863; 2w-ANOVA) or main effect of neonatal intervention were detected (*F*_(2,30)_ = 0.471, *p* = 0.629; 2w-ANOVA), which as mentioned before, suggests no significant differences in GABA_A_R content at dHc synapses.

As in the BLA, no significant interaction (*F*_(2,30)_ = 0.078, *p* = 0.925; 2w-ANOVA) or main effects (neonatal intervention: *F*_(2,30)_ = 0.718, *p* = 0.496; reactivation: *F*_(1,30)_ = 0.045, *p* = 0.833; 2w-ANOVA) were found for the GluN2A/GluN2B synaptic ratio (data not shown).

### k48-Linked Polyubiquitin Levels Were Increased by Reactivation in the BLA, but Not in dHc

k48-linked polyubiquitination was assessed 60 min after memory reactivation since it has been demonstrated that at this time point, retrieval-induced UPS activation significantly increases both in the hippocampus and amygdala (Lee et al., 2008; Jarome et al., 2011). A significant increase was detected in Reactivation animals in the BLA (Figures 4E,F; *F*_(1,31)_ = 5.041, *p* = 0.032, 2w-ANOVA, main effect of Reactivation, no interaction). In the dHc, no interaction or main effects were detected (Figures 8D,E; *p* > 0.05, 2w-ANOVA). k48-linked polyubiquitination is an indirect measure of protein degradation, which in the amygdala has been successfully correlated with memory destabilization (Jarome et al., 2011, 2016; Jarome and Helmstetter, 2014), but in the hippocampus, more direct techniques have been used to link protein degradation to retrieval-induced memory destabilization, including UPS inhibition (Artinian et al., 2008; Lee et al., 2008; Sol Fustiñana et al., 2014) and sample purification using the 26S proteasome subunit S5a before Western blot experiments (Lee et al., 2008), so it is possible that the method used here may lack sensitivity to detect hippocampal protein degradation.

## Discussion

Neonatal interventions change memory consolidation and retrieval (Kosten et al., 2012). Here, we showed that they also affect aversive memory reconsolidation. Unlike NH rats, H and MS animals showed resistance to reconsolidation disruption by mdz, a GABAergic drug, administered after memory retrieval. At a molecular level, both groups showed changes similar to NH animals in the BLA after memory reactivation, but their dHc appeared to respond differently. Furthermore, behavioral expression of the aversive memory was very different in the two groups: while H animals exhibited significant less freezing to the conditioned context presentation, MS rats generalized their fear response to a new and unconditioned context.

Decreased retrieval-induced freezing shown by H rats is consistent with reduced emotional reactivity and the proposed increased inhibitory control of the amygdala by the medial pre-frontal cortex (Stevenson et al., 2008), since amygdalar nuclei are involved in fear expression through its excitatory projections to the periaqueductal gray—PAG (Gross and Canteras, 2012). It could also be the result of cortical or hippocampal dysfunction and consequently impairments in context recognition. However, H males perform normally in memory tasks that are associated with neutral or appetitive stimuli (Kosten et al., 2007; Noschang et al., 2010, 2012) so the cognitive impairment hypothesis has been disregarded (Kosten et al., 2012). Furthermore, re-exposure to the aversive context is stressful for H animals, as can be seen by corticosterone secretion 15 min post exposure (Couto-Pereira et al., 2016), pointing that these animals are perfectly able to recognize the danger and the associated context. Besides, several studies have pointed out that H animals exhibit a behavioral and neurochemical profile of resilience to stress, when adults (Plotsky et al., 2005; Meaney et al., 2013). Together with the low freezing shown here, it is reasonable to assume that the fear memory acquired by H animals may be less emotionally aversive, which may have affected its consolidation and, consequently, reconsolidation.

Aversive memory generalization is a less investigated type of memory impairment, which can be observed when animals freeze to unspecific cues. This trait has often been associated with PTSD, both in animal models and clinical studies (Xu and Südhof, 2013; Thome et al., 2018). Fear generalization to a new context has already been shown in the adult neonatal isolation offspring and was attributed to enhanced theta synchronization in the hippocampus–amygdala–cortical loop during REM sleep by the authors (Sampath et al., 2014). MS rats in our study exhibited higher freezing in the new context (B), compared to H, which suggests fear memory generalization (Winocur et al., 2009; Yang et al., 2011; Sampath et al., 2014). Novelty anxiety impact on this result was discarded by showing that before receiving footshocks, MS animals did not freeze more than the other groups in an unfamiliar environment. A previous study from our group has shown that a strong aversive experience subsequently impairs spatial memory in MS rats (Diehl et al., 2012), which suggests that stressful events may impair MS hippocampal function, causing the animals to have lower ability to discriminate contextual cues, a feature that requires the hippocampus (Fanselow and Dong, 2010), and setting them to exhibit a PTSD-like phenotype (Finsterwald et al., 2015). The absence of behavioral differences comparing MS rats to the other groups before the stressful event (footshock), followed by fear generalization after being exposed to a stressor is in accordance with the second-hit hypothesis (Daskalakis et al., 2013; Finsterwald et al., 2015).

Since H and MS rats showed resistance to mdz interfering effect on memory reconsolidation, we further investigated the molecular pathways involved with this process in the BLA and dHc. Both these brain regions are involved with the processing of contextual aversive memories, as pointed earlier (Phillips and LeDoux, 1992), but operate in an unequal manner (Cammarota et al., 2008). ERK 1/2 signaling has been differently implicated in the molecular mechanism of memory destabilization and reconsolidation in the dHc and BLA (Tronson and Taylor, 2007; Besnard et al., 2013, 2014). Here, we reported that 15 min after reactivation, ERK 1/2 activation was altered in the dHc, but not in the BLA, possibly because in the latter, increased pERK 1/2 levels have been reported only 30 min after reactivation (Besnard et al., 2014). Results presented here refer to cytosolic protein levels. Upon activation, pERK 1/2 translocates to the nucleus, where it phosphorylates downstream transcription factors (Treisman, 1996), which has been shown to be essential for LTP in the dentate gyrus (Davis et al., 2000). Hence, it is reasonable to hypothesize that at the time point analyzed here, in NH animals, activated ERK 1/2 could have already migrated to the nucleus, causing the observed lower levels in the cyt fraction, while in MS rats, this process was still beginning since differences in absolute and relative pERK 1/2 levels were found in these animals. To further elucidate this hypothesis, it would be interesting to analyze the activation of its nuclear substrates, such as the cAMP response element-binding protein (CREB) or Elk-1.

Zif268, an inducible transcription factor which expression is also regulated by ERK 1/2 signaling (Davis et al., 2000; Tronson and Taylor, 2007), is necessary for reconsolidation (Bozon et al., 2003; Lee et al., 2004; Besnard et al., 2013), and has been repeatedly found increased in the hippocampus (Hall et al., 2001; Besnard et al., 2014) and amygdala (Hall et al., 2001; Maddox et al., 2011; Espejo et al., 2016), during contextual memory reconsolidation. We reported here a significant increase in Zif268, 60 min after the reactivation session in the BLA of all animals, independently of the neonatal treatment. In contrast, in the dHc, only NH rats showed a significant increase in Zif268 levels, but not H or MS animals.

Increases in GluN2A, GluN2B and its phosphorylated form were also observed in the BLA synap fraction, 60 min after reactivation; whether these changes result from increased synthesis or increased trafficking of receptor subunits to the synapse or both remains unanswered; it does not seem to result from decreased endocytosis, since no changes in the ratio of pGluN2B to total GluN2B were found. Interestingly, an increase in GABA_A_R α1–6 subunits was also found in the BLA after memory reactivation, which to our knowledge, had not been reported previously. Structural and functional changes in postsynaptic terminals of MS rats BLA have been reported, including dendrite hypertrophy and increased spine density (Koe et al., 2016), as well as increased firing rate in the BLA *in vivo*, when a GABA_A_R inverse agonist was administered (Stevenson et al., 2008), so we analyzed synaptophysin levels in the BLA. In the present study, no significant differences were found concerning synaptophysin in the BLA, which is in accordance with a previous study in MS rats of similar age (Andersen and Teicher, 2004); this suggests that there are no marked changes in presynaptic terminals in the BLA of NH, H and MS adult rats. Still, naïve H animals had higher levels of GluN2A compared to NH in the BLA, which appeared to level with controls after consolidation. Receptor composition reorganization is part of the consolidation plasticity process (Kopp et al., 2007), thus it is possible that consolidation in H occurred differently at the BLA, thus subsequently affecting reconsolidation. This hypothesis deserves further investigation, as it may lead to the elucidation of another aspect of resilience induced by early handling.

In the dHc synapt, no changes in NMDAR or GABA_A_R subunits were found. NMDARs in the dHc are crucial for aversive memory reconsolidation; intra-dHc administration of an NMDA antagonist prevented the reconsolidation-induced update of an aversive memory (Crestani et al., 2015; Haubrich et al., 2015) and NMDA subunit composition determined destabilization and restabilization processes (Milton et al., 2013). However, changes in NMDAR activity unrelated to subunit immunocontent cannot be ruled out by our findings.

Memory reactivation by exposure to contextual cues brings the trace back to a labile state which has been attributed to protein degradation *via* UPS (Lee et al., 2008; Jarome et al., 2011, 2016; Sol Fustiñana et al., 2014). Retrieval-induced UPS activation depends on NMDAR-mediated calcium influx and subsequent activation of CaMKII, in the amygdala (Jarome et al., 2011, 2016). A significant increase in the levels of polyubiquitinated k48-linked proteins was found 60 min after retrieval, in the BLA of all groups studied here. Ubiquitin polymeric chains linked through lysine residue 48 are involved with targeting proteins for degradation by the UPS (Mattiroli and Sixma, 2014) and the amount of k48-polyubiquitinated proteins detected in the amygdala have been shown to be correlated with proteasome activity in the amygdala (Jarome et al., 2011). This suggests that memory destabilization occurred in the BLA of all groups. The involvement of protein degradation in memory destabilization has been less studied in the hippocampus; while, like in the BLA, it has been suggested as the mechanism underlying memory destabilization (Lee et al., 2008), inhibition of the UPS in hippocampal areas produced the same effects as protein synthesis inhibitors in spatial memory (Artinian et al., 2008), raising some questions regarding the role and time of hippocampal protein degradation in memory reconsolidation (Jarome and Helmstetter, 2014). Here, we could not detect any significant changes in k48-linked polyubiquitin levels in the dHc, which could also be due to the sensitivity of the methodology used.

So, why did H and MS animals fail to change their behavioral responses to the aversive context after receiving the GABAergic drug mdz, following memory retrieval? The simplest explanation is that the GABAergic system is altered in these animals so that the drug does not achieve the same effect as in NH rats. In accordance, stressful experiences in rats can change GABAergic transmission in the BLA afferents and internal circuits (Caldji et al., 2000; Rodríguez-Manzanares et al., 2005; Stevenson et al., 2008) and may decrease the effect of mdz on memory reconsolidation (Ortiz et al., 2015). In adult H males, increased binding of a non-BZD GABA_A_R agonist was reported in amygdala nuclei, compared to NH and MS (Caldji et al., 2000). In the dentate gyrus of adult animals that were exposed to a single MS event, decreased neuronal GABA_A_R-mediated inhibitory currents were found, as well as changes in α subunits transcription (Hsu et al., 2003). Our findings suggest no differences in the total GABA_A_R density at the synapses of NH, H and MS rats; furthermore, if altered GABAergic transmission was the main explanation, at least H rats would be expected to respond to mdz treatment, since evidences point to an increase in overall GABAergic function in their amygdala. The possibility of a floor effect is not excluded, since H rats exhibited low freezing in all sessions, equivalent to NH freezing after memory impairment by mdz. Hence, it would be interesting to test reconsolidation impairment in these animals in response to a different drug.

However, more relevant to answer this question is that our results suggest that the BLA of H and MS rats underwent a process very similar to what is currently believed to occur during reconsolidation, in terms of Zif268 upregulation, GluN2B subunit and actin increased density at the synaptic membrane and increased protein polyubiquitination, but not their dHc, which showed relevant differences compared to NH animals, particularly regarding the delay in ERK 1/2 activation and the absence of Zif268 induction.

Most studies on reconsolidation boundary conditions have identified impairments in the retrieval-reconsolidation process at the BLA (Wang et al., 2009; Ortiz et al., 2015; Espejo et al., 2016, 2017). In fact, downregulation of GluN2B (Wang et al., 2009) or increased GluN2A/GluN2B ratio (Holehonnur et al., 2016) in the BLA has been proposed to be the mechanism that prevents strong memories from becoming labile and undergoing reconsolidation. Here, no differences were found regarding the ratio of the two subunits. Also, Zif268 increased expression has been shown to be dependent on NMDARs activity (Malkani and Rosen, 2001; Mokin and Keifer, 2005) and H and MS animals had an increase in the levels of this transcription factor. It is plausible to think that despite the proposed differences in basal and stress-induced excitability of the amygdala resulting from different early experiences (Stevenson et al., 2008; Koe et al., 2016), the BLA of NH, H and MS appears to similarly engage in reconsolidation after retrieval of a contextual fear memory. Hence, our results point to the hippocampus or to its interaction with the amygdala as the possible origin of their differences in memory reconsolidation. In fact, in another study in which rats were subjected to a strong and prolonged stress as adults, differences in the hippocampal response were also found in memory reconsolidation resistance (Hoffman et al., 2015).

The dHc is responsible for detecting novelty in the context where memory is retrieved (Rossato et al., 2007) and a mismatch between the expectation the animal has when it is exposed to the context and reality is a condition that has been shown to be necessary to trigger reconsolidation (Pedreira et al., 2004; Agustina López et al., 2016). Futhermore, hippocampal plasticity mechanisms have been implicated in memory update after retrieval (de Oliveira Alvares et al., 2013; Crestani et al., 2015; Haubrich et al., 2015) and blocking protein synthesis in the dHc after memory retrieval impaired subsequent freezing to multiple contextual cues, while the same procedure in the BLA only impaired freezing to an auditory cue (Yang et al., 2011). The BLA-dHc circuit presents a dual-dynamic interaction (Richter-Levin and Akirav, 2000) and orchestrated processing by the two structures in memory reconsolidation has been reported (Wang et al., 2009; Besnard et al., 2014), including enhanced theta synchronization in this circuit during retrieval (Seidenbecher et al., 2003; Narayanan et al., 2007). If impaired or delayed dHc plasticity was the mechanism responsible for the failure in memory update in H and MS rats, it could be the result of differential BLA modulation of dHc in H and MS rats or partial failure in enhancing theta synchronization between the two structures during reactivation. The apparent timeshift in dHc ERK 1/2 activation in H and MS rats supports this hypothesis. The hippocampus has been suggested as a central structure in PTSD (Maren et al., 2013; Abdallah et al., 2017) and aberrant context processing could, in fact, be a mechanism underlying PTSD (Liberzon and Abelson, 2016), possibly more important than amygdala hyperactivation (Diamond and Zoladz, 2015).

In summary, our results suggest that neonatal interventions in rodents are interesting models to study the mechanisms underlying resistance to reconsolidation; they also contribute to the study of dHc-BLA interaction during memory reconsolidation and to the idea that a fine synchrony between brain structures must occur for memory to be labilized; finally, understanding how early experiences, particularly MS, modulate fear memory reconsolidation in rodents may provide interesting insights on the neurobiological mechanisms of PTSD as well as new therapeutical approaches.

## Ethics Statement

This study was carried out in accordance with the recommendations of the Brazilian Law regarding the use of animals (Federal Law 11.794/2008) and the Guidelines for the Care and Use of Mammals in Neuroscience and Behavioral Research (National Research Council 2003). The protocol was approved by the institutional Research Ethics Committee: CEUA-UFRGS, under the number #23844.

## Author Contributions

NC-P, CD, JQ and VM conceived and planned the experiments. NC-P, CLam, AV, CLaz and GK carried out the behavioral experiments. NC-P, CLam and PE performed sample preparation and biochemical experiments. NC-P, CLam, CD, JQ and VM contributed to the interpretation of the results. NC-P wrote the manuscript. All authors provided critical feedback on the manuscript.

## Conflict of Interest Statement

The authors declare that the research was conducted in the absence of any commercial or financial relationships that could be construed as a potential conflict of interest.
